# Measurements of groundwater, hydrodynamics, and sand characteristics at a dissipative sea turtle nesting beach

**DOI:** 10.1038/s41597-025-04455-5

**Published:** 2025-01-21

**Authors:** Jakob C. Christiaanse, José A. A. Antolínez, Meye J. van der Grinten, Falco Taal, Jens Figlus, Timothy M. Dellapenna, Benjamin Ritt, Christopher D. Marshall, Peter A. Tereszkiewicz, Nicholas Cohn, Edward J. Majzlik, Ad J. H. M. Reniers

**Affiliations:** 1 Department of Hydraulic Engineering, Delft University of Technology, Delft, Netherlands; 2 Department of Ocean Engineering, Texas A&M University, Galveston, TX USA; 3 Department of Marine and Coastal Environmental Science, Texas A&M University at Galveston, Galveston, TX USA; 4 Gulf Center for Sea Turtle Research, Department of Marine Biology, Texas A&M University at Galveston, Galveston, TX USA; 5 Department of Ecology and Conservation Biology, Texas A&M University, College Station, TX USA; 6 Coastal and Hydraulics Laboratory, US Army Engineer Research and Development Center, Vicksburg, MS USA; 7 Coastal and Hydraulics Laboratory – Field Research Facility, US Army Engineer Research and Development Center, Duck, NC USA; 8 Echo Ocean Science LLC, Houston, TX USA

**Keywords:** Physical oceanography, Civil engineering, Hydrology, Physical oceanography

## Abstract

Beach groundwater and nearshore hydrodynamic data were collected during a field experiment along two dissipative beach transects on Galveston Island, Texas, in the fall of 2023. The monitored beaches serve as nesting habitat for the critically endangered Kemp’s ridley sea turtle. Conditions ranged from calm to stormy, with two storms occurring during the experiment, inundating the entire beach up to the dune toe. Collected hydrodynamic data include readings from pressure loggers submerged in the foreshore and mounted in groundwater wells in the backshore, data from two wave buoys about 1.5 km offshore, and GoPro timestacks of the instantaneous waterline (wave runup). Other collected data include bathymetry and topography surveys, subsurface temperature and moisture content readings, and sediment characteristics. This comprehensive dataset can be used to (1) study relevant beach inundation and groundwater processes, including their effect on the local ecosystem (e.g., repeated flooding of sea turtle nests), (2) study the propagation of nearshore hydrodynamic processes into the beach matrix and groundwater table, and (3) validate existing beach groundwater models.

## Background & Summary

Sandy beaches serve important societal and ecological functions, not only as natural barriers protecting the inhabited coastal zone from ocean hazards, but also as habitats for a diverse range of species (e.g., nesting sea turtles and marine birds)^1^. However, beaches are under increasing pressure due to climate change and human activity^2^. Rising sea levels and more frequent and intense extreme weather events not only lead to increased overland flooding^2–4^ but also alter groundwater dynamics^5–7^.

Groundwater dynamics play a pivotal role in coastal ecosystem functions as they influence habitat health^8^, salt intrusion^9^, coastal flooding^10^, and sediment transport dynamics^11,12^. The groundwater table is expected to follow sea level rise rates^6,13^ and is above mean sea level under normal circumstances (overheight)^14^. There can be considerable short-term variability in the groundwater table (>1 m) due to high water level events, wave runup, and precipitation^7^. A higher mean ground water table may compound the effects of these short term events. For example, higher beach ground water levels have been reported to increase wave runup^15^. These challenges are especially critical in low-lying habitats like barrier island systems^7,8^ and for endangered species like sea turtles, who depend on sandy beaches for nesting: turtle nests, buried in the sand, require a relatively narrow temperature and moisture window during their 6–8 week incubation period^16^, and are therefore vulnerable to inundation from overland flooding and groundwater^17,18^.

To continuously assess these risks and evaluate potential solutions, researchers and engineers rely on existing knowledge and numerical models to predict beach groundwater dynamics. However, groundwater dynamics are influenced by a complex interplay of factors, including beach slope^14,19^, sediment size and permeability^14,20,21^, and hydrodynamic forcing^14,15^. Not all of these processes are fully understood, and many existing beach groundwater models (e.g.,^15,20,22^) have not been validated against extensive field data across a variety of beach environments. While multiple studies report field data, relatively few combine observations of groundwater and swash^23^, and the data are often not readily accessible (e.g.,^20,24^). Therefore, enhancing our understanding of these processes through field observations is crucial for reducing uncertainty in model predictions and developing strategies to restore and preserve beach habitats effectively.

**Fig. 1:**
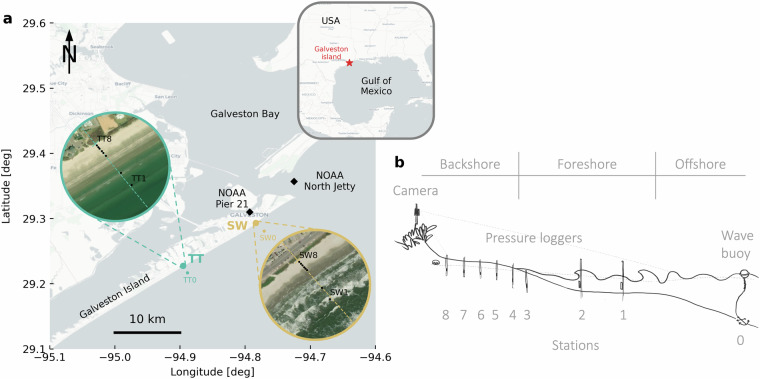
Overview of the field experiments. **a**) Geographical overview of the area around Galveston Island, including the two field sites and NOAA stations. Satellite snapshots show the two field site transects with stations 1–8. Station 0 (offshore) is shown on the large map. **b**) conceptual illustration of the experimental setup at each transect (not to scale). At offshore station 0 a wave buoy measures the incoming wave conditions. Foreshore stations 1 and 2 are equipped with submerged PLs. Backshore stations 3 to 8 are equipped with PLs in groundwater wells and TLs. Basemaps are from OpenStreetMap (www.openstreetmap.org/copyright) and satellite snapshots from ESRI World Imagery (Source: Esri, Maxar, Earthstar Geographics, and the GIS User Community, https://services.arcgisonline.com/ArcGIS/rest/services/World_Imagery/MapServer).

Here, we present data collected during a field campaign in the fall of 2023 at two beach transects on Galveston Island, Texas (Gulf of Mexico). These dissipative, fine-grained beaches^25^ serve as nesting habitat for many marine species, among which the critically endangered Kemp’s ridley sea turtle (*Lepidochelys kempii*)^26^. We measured hydrodynamic processes in the foreshore and groundwater dynamics in the backshore over a period of 1.5 months, using an array of eight pressure loggers per transect—two submerged in the foreshore and six mounted in groundwater wells between the high tide line and the dune toe (Fig. 1). Offshore wave conditions were recorded with two directional wave buoys. These observations were accompanied on several days by GoPro video footage of the instantaneous shoreline (wave runup). We also recorded the sand temperature at a moderate potential turtle nest depth (≈40 cm) next to each groundwater well and a vertical profile of sand temperature and moisture content near the dune toe (where most turtles nest). Finally, we carried out multiple bathymetric and topographic surveys and derived sediment characteristics from extracted cores.

**Fig. 2:**
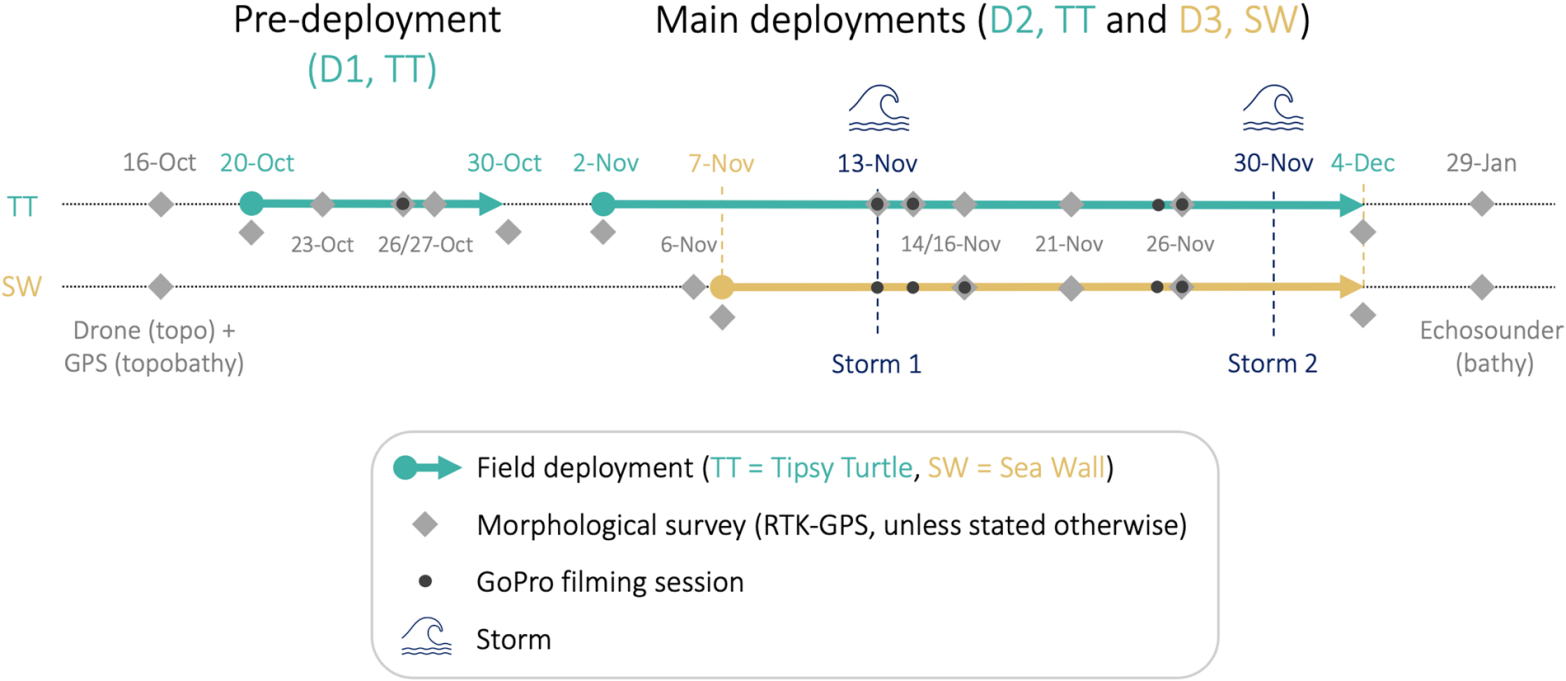
Timeline of the field campaign. The two dotted backround lines represent the timelines at the two field sites. Each deployment period is represented by an arrow. Important dates or surveys are highlighted with labels: morphological surveys (grey diamonds), GoPro sessions (black dots), and the two storms (wave icon). The timeline is not to scale.

All data were collected between 16 October 2023 and 29 January 2024 (Fig. 2) and covered a range of different conditions. Two storms occurred with high waves and water levels inundating the backshore and wave runup reaching up to the dune toe. During these storms, the groundwater head at the dune toe reached the sand surface for several hours. We also observed calm conditions and low water levels during consistent offshore winds. Significant wave heights at the wave buoys ranged from 0.1 to 1.8 m and the total mean water level in the foreshore from −0.4 to 1.2 m NAVD88. This comprehensive dataset can be used to (1) study relevant groundwater and inundation processes, including their effect on the beach ecosystem (e.g., repeated flooding of sea turtle nests), (2) study the propagation of nearshore hydrodynamic processes into the beach matrix and groundwater level, and (3) validate existing beach groundwater models.

## Methods

### Study area

Galveston Island, situated along the upper Texas Gulf coast, is a sandy barrier island which shelters Galveston Bay from the Gulf of Mexico. This large bay connects the Houston metropolitan area to the ocean. Galveston Island stretches southwest for about 45 km from the bay entrance and is fronted by sandy beaches across its entire length, with dunes of about 1–3 m height. It serves as an important habitat for many plants and animals, including two species of sea turtles—Kemp’s ridley and loggerhead (*Caretta caretta*, only sporadically)^26,27^. The Kemp’s is critically endangered and typically only nests along the Gulf of Mexico and southeast US coasts. In the southwest, a naturally stable tidal inlet (San Luis Pass) separates Galveston Island from Follet’s Island. At the northeastern end of Galveston Island, a 3.5-km-long jetty protects the shipping channel into the bay. The city of Galveston is located on the northern part of the island and is protected from the sea by a 17-km-long concrete seawall, constructed in the early 1900s^28^, which has essentially replaced the dunes behind the beach.

General circulation patterns along the upper Texas coast are strongly related to the larger-scale current patterns in the Gulf of Mexico and exhibit a seasonal variation, with a mean northeast bound alongshore current in summer and southwest bound for the remainder of the year^29^. This coincides with the predominant seasonal wave directions (south in spring/summer and southeast in fall/winter), although wave heights are generally low (mean *H*_*s*_ < 1 m^30^). Though Galveston Island has a micro-tidal, mixed-diurnal tide (mean spring tidal range  ≈ 0.8 m^31^), local water levels are very dynamic and regularly differ from the astronomical tide by a factor of two. This is expected to be (partly) caused by atmospheric pressure variations and wind- and wave-induced setup^32^. Furthermore, the region has experienced high sea-level rise rates in the past century (6.5 mm/year since 1904) and is prone to hurricanes during the Atlantic hurricane season (June–November)^33^.

Galveston Island’s beaches are mild-sloping and fine-grained (*D*_50_ ≈ 100–160 μm)^34,35^. In front of the city, they are interrupted by groins every 300–500 m and are regularly nourished, most recently in 2017 and 2019 with dredged material from the bay entrance channel, which is significantly coarser than the native sediment^34,36^. South of the city, there have not been regular nourishments. The combination of the seawall and groins has starved the southwest-bound sediment supply and led to significant erosion southwest of the city in the past decades (up to several meters per year)^33,37^. The coastline in front of the city is generally stable or extending seaward, though mainly due to the nourishments^33^.

### Experimental setup

The experiments were conducted at two field sites along Galveston Island’s gulf coast: Tipsy Turtle (TT) located about 10 km southwest of Galveston (in front of the Tipsy Turtle Sea Bar & Grill, hence the name); and Sea Wall (SW) located in Galveston, in front of the sea wall between 18th and 19th Street. Location TT represents a more natural beach system, with no recent nourishments, whereas location SW is in front of the city, where nourishments have been more frequent.

The field campaign consisted of three deployments (Fig. 2): one pre-deployment at TT from 20 to 30 October 2023 (D1, 10 days) and two main deployments from 2 November to 4 December 2023 at TT (D2, 32 days) and from 7 November to 4 December 2023 at SW (D3, 27 days). The two main deployments (D2 and D3) had the same experimental base design, consisting of nine measurement stations spread over the cross-shore profile, with the most seaward station  ≈1.5 km offshore and the other eight stations in the fore- and backshore (Fig. 1). The stations were named by their field site abbreviation, followed by their number, starting from 0 at the offshore station to 8 at the landward end (dune toe). The pre-deployment (D1) had no offshore station, and its stations were distinguished from D2 by adding an extra 0. So, the stations range from TT10 to TT80 (D1), TT0 to TT8 (D2) and SW0 to SW8 (D3).

At station 0 (offshore) a directional wave buoy recorded the incoming wave conditions (only D2 and D3). Stations 1 and 2, located in the foreshore, were equipped with submerged pressure loggers (PL) to measure incoming waves and water levels. Stations 3 to 8 were spread over the (normally) emerged backshore, roughly between the high tide line and the dune toe, and equipped with PLs, mounted inside 1.5-m-deep groundwater wells, and temperature loggers (TL) buried in the sand at a typical turtle nesting depth (≈40 cm). At station 8 (dune toe) we also installed a vertical array of six buried moisture, temperature, and electrical conductivity loggers, spaced at  ≈10-cm depth intervals, as well as a barometer recording air pressure and temperature (only D2 and D3). At stations 3 (high tide line) and 8 (dune toe) we extracted 1.5-m-deep sediment cores for analysis in the lab. On filming days, the GoPro camera was mounted on a pole near the dune, overlooking the transect and focused on the instantaneous waterline.

We conducted a total of 24 morphological surveys over both field sites (Fig. 2). These included a pre-deployment topographic drone survey (16 October 2023) and a post-deployment bathymetric echo-sounder survey (29 January 2024) of both sites. The remaining 20 were standard walking RTK-GPS beach profile surveys.

### Deployment and instrumentation

**Fig. 3:**
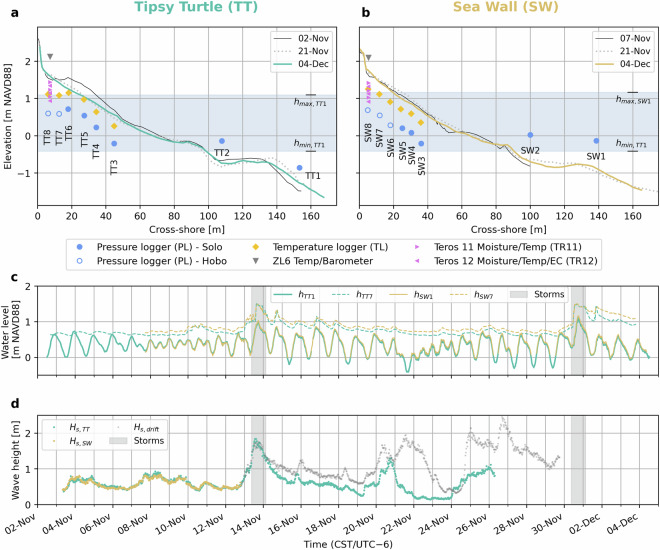
Experimental setup for the two main deployments (D2 and D3). D2 at TT is represented in teal and D3 at SW in beige. **a,** **b**) Cross-shore profiles and instrumentation; **c**) 30-minute averaged water level recorded in the foreshore (*h*_*TT*1_/*h*_*SW*1_) and near the dune toe (*h*_*TT*7_/*h*_*SW*7_); **d**) 30-minute significant spectral wave height recorded by the two wave buoys. Data from the SW buoy after it started drifting is shown in grey.

This section describes the instruments used during the experiments and how, when, and where they were deployed. This is done per instrument/data type. The instrumentation of deployments D2 and D3 is visualized in detail in Fig. 3, along with timeseries of the measured nearshore water level, backshore groundwater table, and wave heights.

#### GPS and morphological surveys

Transect elevation profiles and instrument positions were recorded with handheld RTK-GPS equipment (*Leica CS15 controller*^38^ and *GS08 rover*^39^), hereafter GPS for brevity. Horizontal coordinates were recorded in meters referenced to the North American Datum of 1983 (NAD83) Texas South Central (EPSG:6587), as easting (x-coordinate) and northing (y-coordinate). Elevations (z-coordinate) were recorded in meters relative to the North American Vertical Datum of 1988 (NAVD88). The GPS estimated the 3D accuracy of each point measurement and only recorded a position once the potential error was below 3.5 cm.

The GPS was used to record a total of 20 cross-shore elevation profiles across the deployments and field sites (13 at TT and 7 at SW, see dates in Fig. 2). Starting from the first foredune, we took point measurements every few meters, depending on the slope. The GPS rover was mounted to a 2-m-long survey pole; hence profile measurements were limited to a water depth of roughly 1.5 to 1.9 m, depending on wave conditions. We timed profile surveys with low water levels as much as possible, but given the variable conditions, the extent of the profiles ranged from 40 to 170 m.

On 16 October, we conducted an aerial topographic survey using a *DJI Phantom 4 RTK* drone equipped with a *1-inch 20MP CMOS* camera (FOV 84° 8.8 mm/24 mm f/2.8-f/11 lens)^40^. The drone was combined with a *DJI D-RTK 2 High Precision GNSS Mobile Station* for accurate georeferencing. The GNSS mobile station was referenced at the National Geodetic Survey AW0617 (TT, approximately 2145 m from the field site) and AW1703 (SW, approximately 65 m from the field site) control benchmarks. The drone images were processed using *Pix4Dmapper* photogrammetry software to create a digital elevation model (DEM) and a 3D polygonal model, which includes texture and color data of the terrain. Next, a photogrammetric point cloud was built, based on the estimated drone positions and aligned images, providing detailed 3D data of the area’s physical features. Additionally, we created an orthomosaic, a georeferenced and seamless image, which offers a comprehensive and accurate visual representation of the surveyed area. The individual photogrammetric techniques applied in this process are not explained in detail here, but for an overview of methods we refer to Colomina & Molina (2014)^41^. Post-processing quality assurance and accuracy testing was done using known horizontal and elevation measurements of other National Geodetic Survey or Galveston City reference markers found in the surveyed area. For both locations a horizontal accuracy of 3.26 cm +/− 1.2 cm and a vertical accuracy of 5.31 cm +/− 1.06 cm were found.

Finally, on January 29, when oceanographic conditions and vessel availability finally allowed, we conducted a bathymetric survey along the two transects with a vessel-mounted echo sounder. The survey covered an  ≈80-m-wide area around each transect from the nearshore (≈1m depth) to about 1 km offshore through nine survey transects (spaced at roughly 10 m alongshore). The middle transect survey was extended to about 2.5 km offshore, well beyond the location of the Spotters. This was done with a *Teledyne Odom Echotrac CV100* single-beam echo sounder^42^ operating at 200 kHz and mounted to the side of a small vessel. The exact position of the echo sounder was calculated from a *SBG Ekinox-E* inertial navigation system^43^, which received RTK-GPS positions from a *Trimble MPS865* GPS receiver^44^. Post-processing of the depth readings included despiking, smoothing, velocity of sound adjustments, and tidal correction and was done using *HYPACK Max* software. For the velocity of sound adjustment we applied sound velocity profiles measured near the two Spotter locations using a *Valeport SWiFT SVP*^45^. Like the GPS profiles, the final seafloor coordinates were recorded as easting (*x*) and northing (*y*), referenced to NAD83 Texas South Central (EPSG:6587), and elevation (*z*), referenced to NAVD88.

#### Pressure loggers (PL)

We used a combination of *RBR Solo*^46^ and *HOBO U20L*^47^ loggers to record pressure. The Solos recorded at 16 Hz, except for three which had a maximum sampling rate of 2 Hz. The Hobos measured at 1/15 Hz (every 15 seconds). We used the higher frequency loggers further seaward, where the water level can vary at smaller timescales than further landward. The distribution of the PLs over the eight fore- and backshore stations of each deployment is summarized in Table 1 and visualized in Fig. 3a,b.

**Table 1:** Overview of the PL distribution over the three deployments.

	1	2	3	4	5	6	7	8
D1	Solo 16	Solo 16	Solo 16	Solo 16	Solo 16	Solo 16	Solo 2	Solo 2
D2	Solo 16	Solo 16	Solo 16	Solo 16	Solo 2	Solo 2	Hobo	Hobo
D3	Solo 16	Solo 16	Solo 16	Solo 16	Solo 2	Hobo	Hobo	Hobo

The columns represent the eight stations in the fore- and backshore and the rows are the three deployments. The number behind ‘Solo’ indicates the sampling rate (16 or 2 Hz). All Hobos measured at 1/15 Hz.

In the foreshore (stations 1 and 2) we installed 16-Hz-Solos, roughly 50 cm above the bed, in a porous PVC casing, mounted to vertical galvanized steel poles, which were driven into the sand using a handheld post driver. Conditions during the deployment of D3 on 7 November were quite energetic, which made it difficult to position the PLs at SW1 and SW2. Therefore, they were mounted slightly higher above the bed. As a consequence, the PL at SW1 emerged during several low tides and did not record the lowest water levels.

**Fig. 4:**
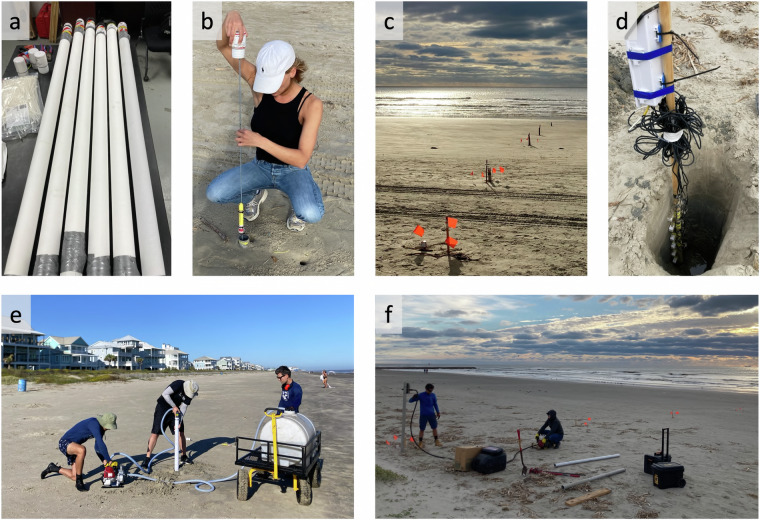
Photo impressions of the field campaign: (**a**) Prepared PVC groundwater wells, wrapped in white filter cloth to limit fine sediment infiltration; (**b**) Mounting of an RBR Solo PL in an installed well. The PL (yellow) is fixed to a metal rod, which is attached to the cap of the well; (**c**) Overview of deployed instruments at TT. Each station is marked with orange warning flags; (**d**) Installation of the Teros and ZL6 loggers at SW; (**e**) jetting installation of one of the wells at TT; (**f**) Extraction of sediment cores at SW using a vibracorer. All photos are courtesy of the authors.

Along the backshore (stations 3–8) the PLs were mounted inside 1.5-m-long slotted PVC wells with an interior diameter of 5 cm and 250 μm horizontal slots along the entire length. Since the slot size was larger than the relatively fine sediment at the beach (*D*_50_ ≈ 150 μm), we used filter cloth to limit the infiltration of fine sediment into the well. For D1 and D2 we tightened polyester filter felt (filtration rating 75 μm) around the wells with tie-wraps every 5 cm and covered the overlap with duct tape. For D3 we used an elastic polyester filter sock (filtration rating 88 μm), which could simply be pulled over the well (Fig. 4a). The wells were sealed with pointed caps at the bottom and flat caps, with a 2-mm-hole for pressure equalization, at the top.

The wells were installed by water-jetting them into the beach surface (Fig. 4e): we sawed off the tip of the pointed bottom cap to create a ≈3-cm-hole, through which we pumped water at high speed using a garden hose and a *Honda WX10T* water pump. This water stream liquefied the surrounding sediment, allowing the well to be pushed into the surface. We stopped when the top of the well was just above the sand surface and pushed a foam plug inside to seal the jetting hole in the bottom cap and prevent sediment from entering the well. The Solo PLs were tie-wrapped to a threaded metal rod which was fixed to the inside of the top well cap (Fig. 4b). To bring them into position we inserted the rod into the well and tightly screwed on the top cap. The Hobos were thicker and not suitable to fix to the metal rod, so we connected them to the well cap through a cord, and installed them by carefully lowering them into the well and screwing on the cap.

Converting the pressure data to a referenced water level required the elevation of each PL. We measured their position with the GPS multiple times during the deployment. Because they were mounted inside wells (backshore) and PVC casings (foreshore) the GPS recordings were indirect, as we measured the position of the top of the well cap and PVC casing respectively. Therefore, we also measured the vertical distance between the sensor of each PL and the GPS measurement point. For the pre-deployment at TT (D1) the PL positions were recorded on 20, 23, 26, 27, and 30 October; for the main deployment at TT (D2) on 2, 13, 16, 21, 25, 26 November and 4 December; and at SW (D3) on 7, 14, 16, 21, 25, 26 November, and 2 and 4 December. However, depending on field conditions, not all loggers could be GPS positioned on every occasion, especially at the foreshore stations.

#### Temperature loggers (TL)

We recorded the sand temperature next to each monitoring well with *HOBO TidbiT MX Temp 400* temperature loggers^48^, measuring at 5-min intervals (1/300 Hz). The TLs were buried in the sand approximately 40 cm below the surface (at deployment). This depth corresponds to the typical nest depth of loggerhead turtles in this region, and was chosen as a representative sea turtle nesting depth since it is deeper than Kemp’s ridley nests, but shallower than loggerhead turtle nests. For D1 we simply buried the TLs and attached them to the wells with a yellow cord to easily retrieve them again. For D2 and D3, we fixed each TL to the bottom of an 80-cm-long wooden stake. The stakes were then buried up to half their length (≈40 cm). This way, the TLs could easily be retrieved, while we marked the stakes with safety tape to serve as visual signals at the field site (as the wells themselves were not easily recognizable).

#### ZL6 and Teros loggers

A vertical array of three *METER TEROS 11 Soil Moisture and Temperature* (TR11) and three *METER TEROS 12 Soil Moisture, Temperature, and Electrical Conductivity* (TR12) loggers^49^ was used to record vertical variations in soil characteristics at station 8 (dune toe). We only had one set available for D2 and D3, so it was deployed at TT8 (D2) from 2 to 21 November and at SW8 (D3) from 21 November to 4 December. The individual TR11/12 loggers were mounted to a wooden pole in alternating order at 10-cm depth intervals, starting with a TR11 (Fig. 4d). They were connected to a *METER ZL6* data logger^50^, mounted above the ground on the same pole. The ZL6 stored the TR11/12 data and recorded local air pressure and temperature, all at 5-min intervals (1/300 Hz). At TT, the array was installed with the first TR11 approximately 10 cm below the surface, the lowest TR12 therefore reaching about 70 cm deep. At SW we installed the array a little bit deeper, reaching from about 30 to 90 cm depth.

#### Wave buoys

At the offshore stations (TT0/SW0) we deployed two *SOFAR Spotter* wave buoys (Spotters)^51^ to record the incident wave conditions during D2 and D3. The Spotter uses satellite GPS to measure its local displacement in three dimensions (XYZ) at a 2.5-Hz sampling rate. From the resulting displacement timeseries it computes directional wave spectra at 30-min intervals^52^. Spotters also record their geographical position (latitude/longitude), the sea surface temperature, relative humidity, and estimated wind velocity and direction every 30 min (the wind parameters are derived from the computed wave spectra^53^). The Spotters were deployed from a vessel on 3 November in a water depth of  ≈8 m (low-tide)—for TT0 this was  ≈1.3 km offshore and for SW0  ≈1.8 km. They were moored to a floating buoy (11.2-liter volume), which in turn was anchored to the seabed through an anchor-chain with five 25-pound weights (total  ≈65 kg for weights plus chain). This indirect mooring setup is recommended by SOFAR, giving the Spotters more freedom of movement compared to direct mooring.

#### GoPro footage

**Fig. 5:**
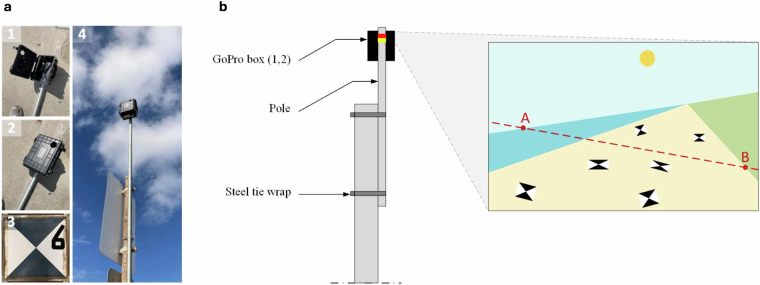
Photos (**a**) and conceptual setup (**b**) of the GoPro cameras to record the instantaneous shoreline. Panel a shows photos of the open (1) and closed (2) protective box, a ground control point (3), and the fully mounted system (4).

The transects were monitored with *GoPro Hero 10 Black* cameras (*23MP CMOS* camera sensor, 16 mm f2.8 lens)^54^ on 26 October (only TT), and 13, 14, 16 (only SW), 25, and 26 November (Fig. 2). The goal was to use the camera footage to extract runup timeseries. Therefore, the cameras were positioned to capture a comprehensive view of the transect, focused on the instantaneous shoreline, while spanning from the distant horizon to the base of the dune. They were securely enclosed within a protective PVC box which was mounted to a pole, approximately 2 m above the ground (Fig. 5). The *GoPro Quick* smartphone app was used for real-time stream access to ensure a satisfactory field view every time they were deployed. To maximize spatial and temporal resolution, the GoPros recorded at 4K with a rate of two frames per second. An external battery was used to prolong the recording time, allowing up to twelve hours of filming. Both cameras were equipped with a 128 GB SD memory card (7–9 hours of filming required approximately 30 GB of local storage).

Intrinsic camera calibration was performed individually for each camera and filming mode to tailor the calibration parameters to specific settings. This involved capturing a short video of a flat checkerboard pattern with known dimensions, under several angles and distances. Radial and tangential distortions were corrected using the OpenCV Python toolbox^55^ along with the camera- and setting-specific calibration matrix, encompassing focal lengths and optical center location. The root mean square reprojection error, which should remain sub-pixel, served as a criterion for the quality of the intrinsic calibration.

Extrinsic camera calibration—georeferencing the two-dimensional undistorted images to real-world coordinates—was done by solving a photogrammetric equation with six unknowns: the real-world coordinates of the camera and the three camera rotation angles (azimuth, tilt, and swing). To approximate the solution we used the CIRN Quantitative Coastal Imaging Toolbox in Matlab^56^. In theory, three ground control points (GCPs) with known real-world coordinates along with an educated guess of the camera position and angles are sufficient to solve the equation. However, in practice, more GCPs are needed for improved accuracy and reliability. We deployed six GCPs during each filming day, strategically positioned above the waterline to cover the entire area of interest and prevent colinearity as much as possible (Fig. 5). During each GoPro deployment, the position of the camera and GCPs was recorded with the GPS.

#### Sediment cores

To analyze the soil characteristics, we extracted two sediment cores from each field site using an *Oztec* vibracorer (Fig. 4f). The cores were extracted at stations TT3/SW3 and TT8/SW8, representing the high-water line and the dune toe, respectively. The cores had a length of approximately 1.5 m and were extracted on 20 October (TT) and 21 November (SW), due to limited availability of the vibracorer. Hence, the SW cores were extracted after the first storm on 13 November, which eroded some of the surface, although the erosion at SW was significantly less than at TT (Fig. 3a,b). After extraction, the cores were sealed and stored in a cool room at 3°C. Between 1 and 4 December, we split the cores in the laboratory and carried out three separate analyses:

- **Grainsize distribution**: We used a *Malvern Mastersizer 2000*^57^ to determine the grain size at 10-cm intervals over the length of each core. At each interval, we extracted a small sediment sample from a 1-cm-thick layer of the core (i.e., between the top and 1 cm from the top, between 10 and 11 cm from the top, and so on). The sample was suspended in water in a small plastic centrifuge tube and then analyzed by the Malvern. To get a more reliable grain size estimate, the Malvern ran each sample three separate times and returned all three measurements, as well as the average.
- **Line-scan imaging**: We created high-resolution line-scan TIFF images of the entire length of the split cores. We used a *Geoscan V* line-scan camera (exposure time 10 ms, calibration aperture 14.12, image aperture 11.39) integrated in a *Geotek Multi-Sensor Core Logger (MSCL)* system^58^. Each core was scanned with a resolution of 200 pixels per centimeter (i.e., 1 pixel = 50 μm).
- **XRF spectrometry**: We conducted X-ray fluorescence (XRF) spectrometry using an *Olympus Vanta XRF* integrated into the same *Geotek MSCL* system as the line-scan^58^. The XRF measured the elemental abundances of a range of elements between Magnesium (Mg) and Uranium (U) every 5 cm.

### Maintenance and disruptions

**Fig. 6:**
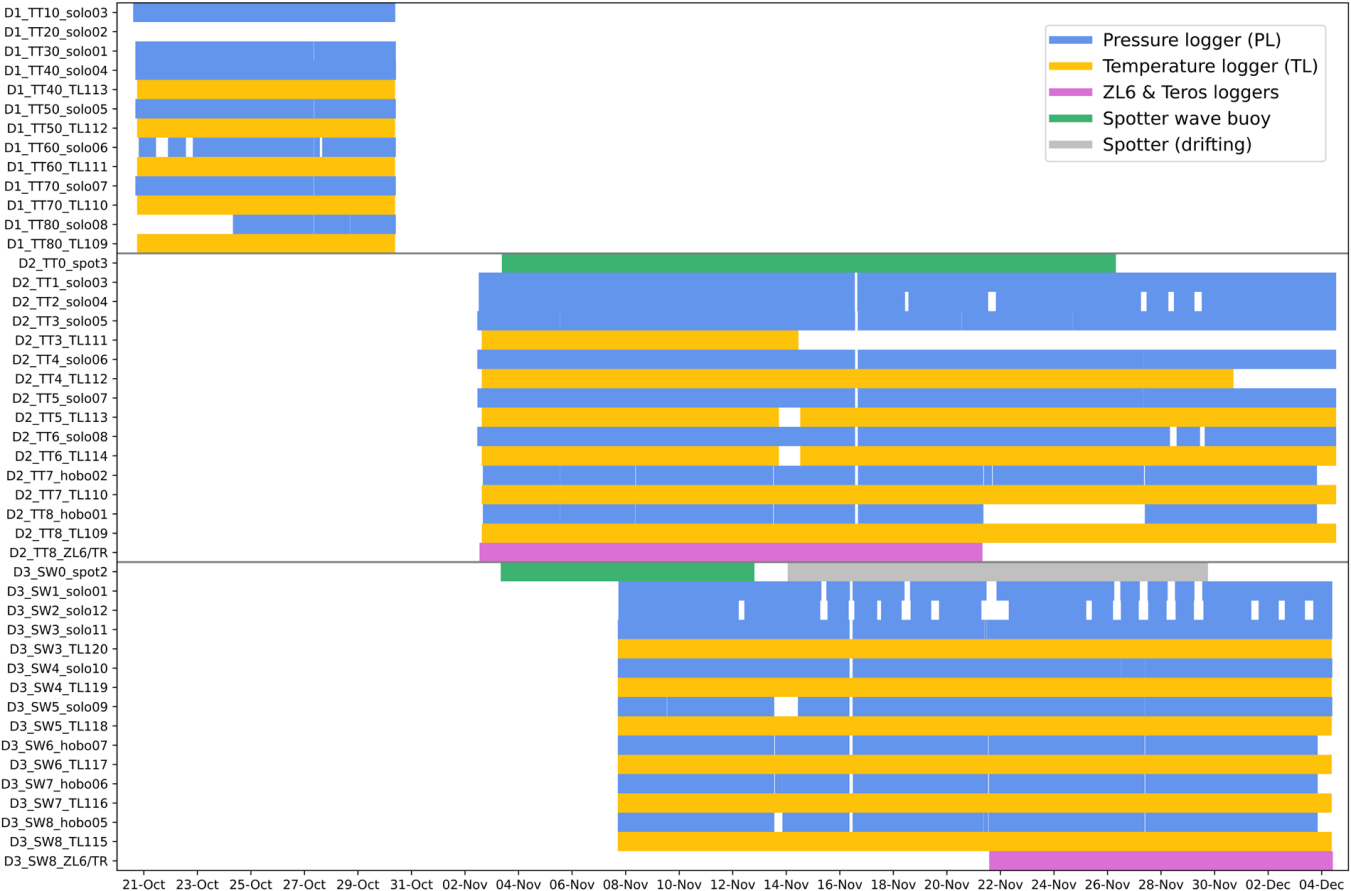
Instrument activity for all three deployments (separated by the grey horizontal lines) of the pressure loggers (blue), temperature loggers (yellow), ZL6/Teros loggers (pink), and wave buoys (green). The ZL6/TR row represents the ZL6 logger as well as all six TR11/12 loggers connected to it.

Several instruments needed some form of maintenance during the fieldwork, causing gaps in the recorded data. Furthermore there were some unforeseen incidents during the deployments, also causing disruptions. In the processed timeseries, these gaps were filled with *Not a Number (NaN)* values. These disruptions are summarized here and an overview of the activity of each instrument, including gaps, is presented in Fig. 6.

#### Pressure loggers

During all deployments we carried out (irregular) inspections of the monitoring wells, quickly unscrewing the cap to check the logger. These quick inspections usually took no more than 1–2 min. Additionally, the Hobo PLs, measuring at 1/15 Hz, reached their internal storage capacity after about six days. Hence, they had to be removed from the well to offload the data at least every six days, which normally took about 15 min. The Solos had enough capacity to record continuously for the entire deployment duration. Nonetheless, on 16 November, we retrieved all deployed PLs (D2 and D3, foreshore and backshore) to offload data. This was done as a precaution to check if anything went wrong and to safely back up the first part of the data. This means that no data were recorded at SW (D3) between 08:45 and 11:30, and at TT (D2) between 13:45 and 16:20 Central Standard Time (CST/UTC–6). Next to these regular maintenance breaks, there were several incidents that led to gaps in the data and/or changes in the vertical position of the PL:

- After the retrieval of the instruments of D1, the PL from TT20 (*solo02*) was unresponsive and could not be read out. No exterior damage was discovered so it was sent to RBR Global for assessment and repair. Unfortunately, no data could be recovered from the logger, so there is no recorded pressure data for D1 at TT20.
- The monitoring well at TT60 (D1) was hit by a truck on 27 October at 12:27 CST (time determined from spike in recorded pressure). Luckily, the PL (*solo06)* was not damaged and kept recording. We replaced the well and reinstalled the PL at 16:15 CST on the same day.
- Similarly, the well at SW5 (D3) was hit by a truck on 9 November at 13:09 CST, however, we only discovered this on 13 November. The PL (*solo09*) was not damaged and kept recording, although during the hit it moved downward by 11 cm. The time and vertical movement were determined from a clear step in the recorded pressure. We removed the PL on 13 November 13:15 CST and replaced the well and reinstalled the PL on 14 November 10:30 CST, which moved the logger upward again by 6 cm.
- On 13 November, during a quick check, we discovered that the plug in the well at SW8 was not weighted correctly and had moved upward around 13:20 CST (time determined from step in pressure data), also pushing the installed PL (*hobo05*) upward (unlike the Solos, which were fixed to a metal rod, the Hobos were hanging on a cord from the well cap). We pushed the plug down again and put extra weight on top and the PL was in position again on 13 November 20:35 CST.
- After the storm on 13 November the steel poles at TT1 and TT2 (D2) were visibly tilted, moving the PLs (*solo03* and *solo04*) slightly downward (more so at TT1). Unfortunately the only pre-storm recording of the PL positions was on 2 November, directly after deployment. After the storm, the positions were recorded on 16 and 21 November and 4 December. The difference between the pre-storm position and the average of the three post-storm ones gives a downward vertical movement of 9.3 cm at TT1 (*solo03)* and 3.7 cm at TT2 (*solo04*), although it is not entirely clear how accurate the pre-storm positions were and if the entire movement was due to the storm. We could not identify a significant step in the recorded pressure series so the movements likely happened gradually during the storm.
- During the data offload on 16 November, we discovered some sand in the lower part of the well at TT3 (D2), which probably entered the well during the storm on 13 November. There were no problems retrieving the PL (*solo05*), however as a precaution we reinstalled it about 16 cm higher up on the metal rod.
- During the data offload of the Hobos at TT (D2) on 27 November we discovered that *hobo01* at TT8 had malfunctioned after the previous offload on 21 November. No data could be recovered for this period, and we replaced it with a spare Hobo for which we kept the same ID (*hobo01*) from 27 November 9:03 CST. We used the same cord so the logger elevation did not change.
- Finally, the PL elevations at TT2, TT6, SW1, and SW2 led the loggers to be emerged during several periods with low water levels. In the foreshore (TT2, SW1, and SW2) this was the case during some of the low tides. In general the PL elevations were chosen based on a trade-off between having enough depth and minimizing the risk of the logger getting buried if it were installed to close to the bed. At SW1 and SW2 the loggers were also installed slightly higher because of the difficult and energetic conditions during deployment, and were not changed afterwards. At TT6 the logger was installed a few cm too high for the lowest water levels on 28 and 29 November, due to the presence of the berm at deployment, resulting in gaps of six and four hours, respectively.

#### Temperature loggers

There was no regular maintenance involved for the TLs, but several disruptions occurred, all of which during D2 at TT. We believe that the reason for this is that the beach at TT eroded significantly more than at SW (up to 40 cm in some places, Fig. 3a), which destabilized the wooden stakes to which the TLs were mounted.

- On 13 November around 17:30 CST, during the first storm, the TLs at TT5 (*TL113*) and TT6 (*TL114*) were washed away (as they were mounted to wooden stakes protruding from the sand surface). The loggers were not damaged and were recovered and reinstalled on 14 November 12:30 CST.
- On 14 November around 11:00 CST the TL at TT3 (*TL111*) disappeared from its position (the reason is unclear). It was found and recovered undamaged in the foredune later in November but not reinstalled.
- On 30 November around 17:00 CST, during the second storm, the TL at TT4 (*TL112*) was washed away. It was recovered undamaged on 1 December but not reinstalled.

#### Wave buoys

Unfortunately, neither Spotter completed the full deployment period. The SW0 Spotter started drifting from its deployment position on 12 November, nine days after its deployment. It is unclear what exactly happened, but it was most likely caught or hit by a vessel or trawling net, as it briefly moved upwind. It then started drifting southward freely on 14 November and kept recording for the next 17 days. It finally stranded on 29 November in Mexico, about 490 km southwest of Galveston, and was recovered, still connected to the secondary floating buoy (but not the anchor chain and weights). The TT0 Spotter stayed in position until 26 November, when it was collected in the net of a local fishing vessel (confirmed by the captain of the vessel). Unfortunately, the crew released the buoy from the vessel a few days later and it ultimately stranded on Matagorda Beach on 30 November, roughly 120 km southwest of Galveston, where it was recovered. During its trip, it lacked the ballast chain (likely removed on the vessel) and therefore the required stability for reliable measurements. Fortunately, both Spotters were recovered intact and with undamaged memory cards. There is no Spotter data from either TT0 or SW0 after 26 November, which includes the second storm on November 30th. However, the data from the SW0 Spotter during its entire trip to Mexico is available (Fig. 3d).

## Data Records

All data are stored open access on the 4TU.ResearchData repository, as a data collection called *TURTLE: Measurements of groundwater, hydrodynamics, sand temperature, and sediment characteristics at two beach transects on Galveston Island, Texas, USA*^59^, accessible through the following link: 10.4121/93256801-ed01-4627-9e49-8607967a0853. The collection includes seven datasets (the peer reviewed version numbers corresponding to this publication are denoted by the v-numbers at the end):

- TURTLE - Main data - deployment D1 - location TT (20–30 Oct 2023), v3, [Link]
- TURTLE - Main data - deployment D2 - location TT (2 Nov–4 Dec 2023), v3, [Link]
- TURTLE - Main data - deployment D3 - location SW (7 Nov–4 Dec 2023), v3, [Link]
- TURTLE - GoPro Timestacks (26 Oct–26 Nov 2023), v2, [Link]
- TURTLE - Beach profiles and bathymetry (16 Oct 2023–29 Jan 2024), v2, [Link]
- TURTLE - Topographic drone survey (16 Oct 2023), v2, [Link]
- TURTLE - Supplementary data, v2, [Link]

Each deployment has its own main dataset, which includes the PL, TL, Spotter, ZL6/TEROS, and sediment core data. The morphological GPS and echo sounder surveys are gathered in the ‘Beach profiles and bathymetry’ dataset. The drone survey data and GoPro timestacks are stored as separate datasets because of their relatively large file sizes. Finally, the supplementary data contain external data that were used in the processing of the observations (i.e., air pressure and water level measured by NOAA^31^).

### Deployment datasets

Each deployment dataset contains all data gathered as part of that specific deployment, except the morphological surveys and GoPro footage. For D1 this is only PL and TL data, for D2 and D3 it also includes the, ZL6/TEROS, Spotter, and sediment core data (even though the cores at TT were extracted on 20 October, the data are gathered in the main data of D2). Each instrument/data type has its own directory within the dataset, where all data files are located, and which also contains a folder called ‘raw’ with the unaltered logger output files (if applicable). Additionally, each folder contains a *README.txt* file with detailed explanations and instructions.

All processed deployment data files follow the same naming convention (except the sediment core data, see below): *YYYYMMDD_YYYYMMDD_AA_SSS_dataname.ext*, where the two *YYYYMMDD* represent the start and end date of the data record, *AA* is the deployment ID (D1/D2/D3), *SSS* is the field site ID (TT/SW) or station ID (TTX/SWX), *dataname* is the data identifier, and *ext* is the file extension. For example, the data from *solo03* at TT1 of D2 are gathered in the NetCDF file *20231102_20231204_D2_TT1_solo03.nc*. Furthermore, for all time series data, the timestamps have been converted to Unix epoch time (seconds since 1 January 1970, UTC).

The first directory, ‘0_Meta’, contains relevant meta data for the instruments of the corresponding deployment. This includes CSV files with the measured GPS positions of each instrument, the pressure offsets for each PL used to correct the raw data (see Technical Validation section), an overview of the bucket tests carried out to validate the PLs, and a matrix containing the instrument activity shown in Fig. 6.

The processed PL time series are gathered in the directory ‘1_PL’, in one NetCDF (*.nc*) file per PL. Each NetCDF file includes five variables: raw pressure (*pr*), processed pressure (*pp*), water depth (*d*), logger elevation (*z*), and water level relative to NAVD88 (*h*). For storage efficiency, the data have been converted to Pascal (pressure) and mm (depth/elevation) and stored as integers, with invalid data points given a value of  –9999. Raw pressure, *pr*, are the raw pressure readings from the logger. The processed pressure, *pp*, is *pr* corrected for the atmospheric pressure at NOAA’s Pier 21 station (ID 8771450, included in supplementary dataset) and, if required, a logger specific pressure offset (see Technical Validation section). Additionally, values at inactive timestamps (e.g., data offload, emergence during low-tide, disruptions) have been filtered and replaced with –9999. The water depth above the logger, *d*, was computed hydrostatically by dividing *pp* by the gravitational acceleration (*g* = 9.81 *m*/*s*^2^) and the density of sea water (*ρ* = 1023.6 *kg*/*m*^3^). The logger elevation, *z*, was derived from the GPS recordings (see Technical Validation section) and the final water level relative to NAVD88, *h*, is the sum of *d* and *z*. Due to their large file sizes, the raw output files of the PLs are gathered in a separate directory, ‘9_PL_raw’, so they can be downloaded separately if desired. These are *.rsk* files for all Solos, which can be read using the *Ruskin* software by RBR; and *.hproj* files for all Hobos, which can be read using the *HOBOware* software from HOBO.

The TL data are gathered in the directory ‘2_TL’. The ‘raw’ folder contains the raw CSV files extracted from each individual TL. The processed time series (converted to Unix epoch time and filtered for inactive timestamps) are gathered in a single CSV file. Its columns represent the measured sand temperature at each corresponding station and are named as *SSS_LOGID*, where *SSS* is the station ID and *LOGID* is the logger ID (e.g., *TT3_TL109* represents the sand temperature at station TT3, which was measured with logger TL109).

The ZL6/TEROS data are gathered in the directory ‘3_ZL6TEROS’. The ‘raw’ folder contains the raw Excel file (.xlsx) extracted from the ZL6 logger. The processed 5-min-interval time series of air temperature (at, ZL6), air pressure (ap, ZL6), volumetric water content (wc, TR11/12), sand temperature (st, TR11/12), and electric conductivity (ec, TR12) are gathered in one CSV file. The variables are named as *Z_LLLL_vv*, where *Z* is the vertical position (starting from 0 for the ZL6 down to 6 for the lowest TR12), *LLLL* is the logger type (ZL6/TR11/TR12), and *vv* is the variable abbreviation (at/ap/wc/st/ec). For instance, *4_TR12_wc* represents the volumetric water content measured by the TR12 at the fourth position from the surface.

The Spotter data are gathered in the directory ‘4_Spotter’. The ‘raw’ folder contains the 15 raw CSV files extracted and compiled from the Spotter SD card, including the full 2.5-Hz displacement time series. For an overview of the data in each raw file we refer to the Spotter GitHub repository^60^. The folder ‘bulk’ contains the 30-min-interval bulk parameters that were transmitted live during the Spotter deployment, including spectral wave parameters, estimated wind velocity and direction, relative humidity, sea surface temperature, and geographic position (latitude/longitude). The folder contains one CSV file each for the raw and processed bulk data. The latter were manually filtered for outliers in the significant wave height and mean wave period (Fig. 3d). All wave parameters at a timestamp with an outlier in either the significant wave height or mean wave period were set to *NaN*. The wind, temperature, humidity, and geographic coordinates were not filtered for outliers, but we did filter data at invalid timestamps at the start and end of the time series for all variables. The CSV file for the SW0 Spotter contains an additional column, *drift_flag*, which was set to 0 during the normal deployment and to 1 for all records gathered when it was drifting (see teal and grey data in Fig. 3d). Invalid data entries in-between were filtered. Due to privacy reasons we removed all location data from before the Spotters were released in the water and after they stranded on the beach (both in the raw and processed files).

The sediment core data are gathered in the directory ‘5_Cores’ of the main datasets of D2 (TT) and D3 (SW). All files are named *YYYYMMDD_SSS_dataname.ext*, where *YYYYMMDD* represents the extraction date of the core, *SSS* the station ID, *dataname* the data identifier, and *ext* the file extension (e.g., *20231121_SW3_grainsize.csv* contains the grain size distributions for the SW3 core). Grain size data are provided in two files per core: the raw TXT file containing the tabled output of the Malvern, and a processed CSV file containing the summarized grain size distributions at each depth interval, based on the average of the three Malvern runs per sample. The line-scan images are provided in four files per core: the raw TIFF image of the entire core, an edited TIFF image with a length-scale along the left side, a raw XML file containing metadata about the core dimensions, camera setting, and resolution, and a raw RGB file. The three raw files can be read together using *Geotek* software^58^. For the XRF we only provide the raw data files: an OUT file containing the tabled output data in text format, and a binary DAT file containing the data and instrument calibrations, which can be read using *Geotek* software.

### Morphological surveys

The morphological survey data is split into two datasets, one containing the beach profile and bathymetric echo sounder data and a separate dataset for the drone survey because of its large file sizes. The first is divided into two directories: *GPS*, containing the handheld GPS profiles, and *Echo*, containing the bathymetric echo sounder survey data. The GPS profiles are provided in one CSV file per recorded beach profile and named *YYYYMMDD_AA_SS.csv*, where *YYYYMMDD* represents the survey date, *AA* the deployment ID (D1/D2/D3), and *SS* the field site ID (TT/SW). The columns in the CSV file contain the timestamp of each point measurement and the horizontal and vertical coordinates, including coordinates in a deployment specific cross-shore coordinate system (see below). The echo sounder survey is provided in two CSV files (one per field site) containing the time and horizontal and vertical coordinates.

The horizontal coordinates of the GPS surveys are given in two official coordinate reference systems: NAD83 Texas South Central easting (e) and northing (n) in meters (EPSG:6587), and WGS84 latitude (lat) and longitude (lon) in degrees (EPSG:4326). The vertical coordinate (z) is in meters relative to NAVD88. To reference all profiles and instrument positions of one deployment to a single cross-shore axis, we created a local, deployment specific coordinate system by fitting a line through the longest and straightest profile of each deployment and defining an origin behind the dune toe (Fig. 3a,b). We then projected all GPS profiles and instrument positions to this line resulting in a cross-shore coordinate, *x*, the distance to the origin, positive in seaward direction. The alongshore coordinate, *y*, represents the distance between the projected and measured points. The local coordinate systems are referenced to real-world coordinates through the axis angle (relative to North) and the coordinates of the origin (Table 2).

**Table 2:** Local cross-shore coordinate system for each deployment, referenced to real-world coordinates through the angle relative to North and the coordinates of the origin (E/N = easting/northing, lon/lat = longitude/latitude).

	Base profile	Angle [°*N*]	Origin E [*m*]	Origin N [*m*]	Origin lon [°]	Origin lat. [°]
D1	20 Oct	142.03	998954.83	4161529.28	−94.89517	29.22765
D2	2 Nov	140.61	998951.14	4161526.83	−94.89521	29.22763
D3	4 Dec	138.22	1009525.55	4169174.65	−94.78367	29.29320

The topographic drone survey data are gathered in five files per field site: two TIFF files for the DEM and the orthomosaic, a LAZ file containing the point cloud, an OBJ file containing the 3D polygonal surface mesh, and an accompanying MAT file which contains material definitions for the polygonal mesh (e.g., color and texture). The latter two can be loaded into CAD software.

### GoPro timestacks

The GoPro timestacks are gathered in a separate dataset and all files are named *YYYYMMDD_HHMM_AA_SS_GXCODE.ext*, where *YYYYMMDD* is the date of the recording, *HHMM* is the starting time in CST, *AA* is the deployment ID, *SS* is the field site ID, and *GXCODE* is the GoPro file ID. For storage efficiency, the GoPros store a long filming session in several separate files, the order of which can be determined from the GoPro file ID. For practical reasons, we named all files with the starting time of their respective session. The timestacks are stored in NetCDF files which contain the RGB values of each pixel, as well as the time and space dimensions. The spatial coordinates are given in easting and northing NAD83 Texas South Central. Additionally, we projected the timestack transects to the deployment specific cross-shore coordinate systems (*x* and *y* coordinates). There is also a folder with plots of each timestack so they can be visually inspected without having to read all the data. Due to the enormous file sizes of the individual videos, we decided not to include them in the data repository. However, the video footage may be acquired by contacting the authors directly.

### Supplementary data

The supplementary dataset contains records of the air pressure recorded at NOAA station *8771450* (Galveston Pier 21) and water level recorded at NOAA station *8771341* (Galveston Bay Entrance, North Jetty). Both were obtained from the NOAA Tides and Currents database^31^ and are provided as CSV files.

## Technical Validation

### Pressure loggers

Atmospheric pressure is continuously recorded at 6-min intervals by NOAA’s Pier 21 station in Galveston (station ID *8771450*, 2 km from SW and 14 km from TT)^31^. The ZL6 logger also measured air pressure at TT8 from 2 to 21 November and at SW8 from 21 November to 4 December. The recorded pressure from the ZL6 compared very well to the NOAA station at both field sites (*r* = 1.00, *RMSE* = 0.21 mbar at TT and *r* = 1.00, *RMSE* = 0.16 mbar at SW).

We let all PLs record the atmospheric pressure for at least 30 min, before and after each deployment, as well as once during the two main deployments (on 16 November). To validate the recorded pressure time series we compared them to the ZL6 and/or NOAA readings. If the mean absolute deviation over the calibration period was larger than 1 mbar (≈1 cm of water under hydrostatic conditions) we corrected the pressure data according to the measured offset. Furthermore, we conducted bucket tests with seawater from the respective field site before and after each deployment, and again on 16 November, during the two main deployments. During these tests, the loggers were simultaneously dipped in a bucket of water. Once the water surface settled from the disturbance, we measured the water level in the bucket. The corrected pressure time series converted to within 1–2 cm of the measured bucket water levels, which we deem to be an acceptable margin. Most of the PLs did not show a significant temporal drift in the recorded offset, so a constant offset was applied to the entire time series. For some loggers the offset was more complicated:

- *solo08* at TT80 (D1) remained emerged for the first days of D1 as the groundwater table did not reach the logger. Until late on 21 October the readings compare well to the air pressure measured by NOAA. However, from then on a negative pressure offset started to develop, growing linearly until 24 October at about 4:50 CST, when a sudden spike increased the pressure by about 5 mbar, moving it back near the recorded pressure by NOAA. During this period the groundwater table was rising (confirmed by the other loggers). The spike was likely caused by the groundwater first touching the logger. Unfortunately, it is not entirely clear whether the pressure offset was still present when the logger was fully submerged. However, after the spike, the recorded pressure was near the NOAA record again, slowly increasing as the groundwater submerged the logger. Additionally, *solo08* recorded the correct water level in both bucket tests. We therefore assumed no pressure offset when the logger was submerged. Given that the ground water table at TT80 cannot be derived from the logger when it was emerged, we filtered all recorded pressure values until 24 October 8:00 CST (when the sensor appeared fully submerged) in the processed data.
- *solo01* showed a positive offset with a temporal drift in both of its deployments (D1 and D3). For D1 it went from  ≈5.5 mbar at the start to  ≈7.5 mbar at the end, and at D3 from  ≈6 mbar at the start to  ≈8 mbar at the end (during the offload on 16 November it was  ≈7 mbar). We therefore linearly interpolated the offset between the determined values on 7 and 16 November and 4 December.
- *A*fter malfunctioning, *hobo01* was replaced by a spare logger, for which we retained *hobo01* as ID. The original *hobo01* had a constant offset of  −2.5 mbar, whereas the new hobo had no offset. This was corrected in the processed time series.
- *solo09* (only deployed for D3 at SW5) appeared to be the most inconsistent logger with varying offsets, albeit always relatively small (<2 mbar). However, in all three bucket tests it recorded the correct water level within 1 cm, so we did not apply any offset to it.
- For the Hobos of D3 (*hobo07* at SW6, *hobo06* at SW7, and *hobo05* at SW8) an offset was found at the time of deployment (7 November), when all three loggers appeared to be 2–3 mbar off (also translating to 2–3 cm in the bucket test). However, at the first data offload (13 November) the air pressures were not off anymore and no offset was found for the remainder of the deployment. Moreover, the difference in corrected pressures between the six groundwater stations did not seem disproportional during the first week. We figure the offset at the start may have been caused by the loggers needing to ‘acclimatize’ to the temperature. Given the relatively small offset and the uncertainty whether it persisted into the first deployment week, we did not correct it in the data (i.e., all three PLs had an offset of 0 over the entire deployment).

### Logger elevations

Every point measurement with the GPS returned an estimate of its 3D accuracy, usually between 2–3.5 cm. Between multiple measurements of a fixed location, the expected range around the true position should therefore be 4–7 cm. This was tested by recording the position of five fixed points on three different days (10, 20, and 24 May 2024), resulting in a mean elevation range of 4 cm (*std* = 1.8 cm). In theory, the more measurements are taken, the more the range should converge to its maximum value and the mean to the true position.

The first GPS recordings of the backshore PLs of D1 and D2 were taken shortly after jetting the groundwater wells into the beach and mounting the loggers. Given the rather intrusive jetting procedure, the soil around the well may have needed some time to settle back to pre-jetting conditions, potentially resulting in a slight movement of the well. This hypothesis is supported by the fact that the first recorded elevation was often at one of the limits of the measured range. We therefore excluded elevation recordings taken on the same day as the jetting when computing the elevation ranges for all backshore PLs (stations 3–8). This was only relevant for D1 and D2, as the wells for D3 were jetted on 6 November, a day before deploying the loggers and recording their positions.

The resulting elevation ranges are mostly below or near the expected 7-cm limit (Table 3). For four PLs, the elevation range was above this limit, namely 9.3 cm at TT40 (D1, *solo04*), 12.2 cm at TT1 (D2, *solo03*), 7.9 cm at SW7 (D3, *hobo06*), and 9.6 cm at SW8 (D3, *hobo05*). The large range at TT1 can be attributed to the tilting of the pole during the storm. However, we could not determine the exact cause for the higher ranges at TT40, SW7, and SW8. It could still be attributable to the uncertainty in the GPS measurement, however it is also plausible that the wells at these stations slightly moved at some point during the deployments. Practically the entire range at SW7 and SW8 can be attributed to a large elevation change between 14 and 16 November. It could be that the wells slightly moved due to something happening in the sand, but it seems unusual for that to happen in only two days’ time and not during either of the storms. Another possibility could be interference from a bypasser, although one would then expect a relatively sudden shift, which we could not identify in either pressure time series.

**Table 3:** Overview of the PL elevation ranges in cm measured with the GPS.

	1	2	3	4	5	6	7	8	mean	std
D1	2.6	3.9	2.7	9.3	4.2	(0.7)	6.2	5.0	4.9	2.3
D2	(12.2)	4.9	4.2	4.1	4.1	5.3	3.2	5.2	4.4	0.8
D3	3.7	5.3	5.7	5.4	5.5	6.2	7.9	9.6	6.2	1.8

Columns 1 to 8 represent the eight stations and the final two columns show the mean and standard deviation over the eight stations. The elevations for TT60 and TT1 (values in brackets at D1/6 and D2/1 respectively) were excluded from the mean and standard deviation. The reason is that they did not reflect appropriate conditions (the PL at TT1 moved during the storm on 13 November, and for TT60 there were only two measurements as the well had to be replaced mid-deployment).

For the final logger elevations, needed to derive the referenced water level from the pressure data, we used the mean of all valid recorded elevations (excluding the first recording directly after jetting, as mentioned above). In case the elevation of a PL changed over time, we adjusted this in the data. The sudden changes were easily corrected with a stepwise elevation profile in time. For the PLs at TT1 and TT2 we applied a linearly interpolated elevation profile to account for the elevation change over the course of the storm on 13 November. We used the only recorded pre-storm elevation (from 2 November) until 13 November at 10:00 CST and the mean post storm elevation as of 13 November at 21:00 CST. In between, we linearly interpolated the elevation.

**Fig. 7:**
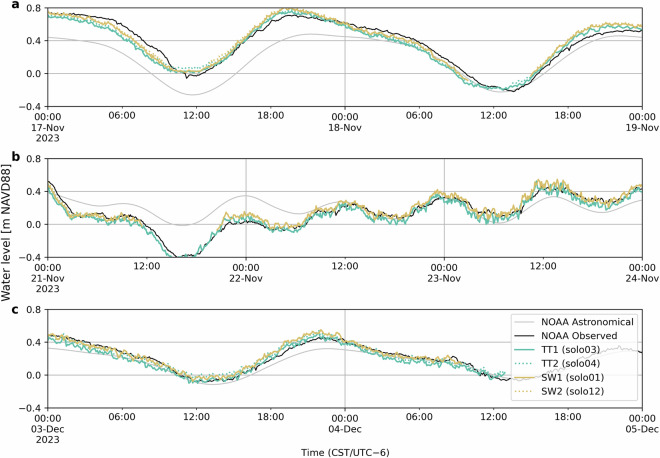
Recorded water levels at TT1 (*solo03*), TT2 (*solo04*), SW1 (*solo01*), SW2 (*solo12*), and NOAA station 8771341 (Galveston Bay Entrance, North Jetty), during three periods of relatively calm conditions (low wind and low waves): 17–18 November (**a**), 21–23 November (**b**), and 3–4 December 2023 (**c**).

For the foreshore PLs (stations 1 and 2) we did an additional validation step by comparing the observed water levels during calm conditions (low wind, low waves). The expected low surge and setup components mean that the water level at the two field sites and NOAA station 8771431 (Galveston Bay Entrance, North Jetty) should be similar. Small differences are still expected, especially compared to the NOAA station, which is in the inlet channel of Galveston Bay (Fig. 1a). We identified three periods of relatively calm conditions, from 17–18 November (*H*_*s*_ < 0.5 m), 21–23 November (*H*_*s*_ < 0.3 m), and 3–4 December. All three periods had generally low residual water levels (i.e., surge), shown by the small difference between the observed water level and the predicted astronomical tide at the NOAA station (Fig. 7). Over the three periods, the difference between the observed water levels at the four field stations (TT1, TT2, SW1, SW2) and NOAA is very similar, with an RMSE around 6 cm for all four stations (Table 4). As expected, the RMSE between stations at the same location was significantly smaller (2.7 cm between TT1 and TT2, and 1.2 cm between SW1 and SW2). The RMSE between the same stations and different field sites (TT1 vs. SW1 and TT2 vs. SW2) was around 5 cm.

**Table 4:** Correlation and RMSE between the recorded water levels at TT1 (*solo03*), TT2 (*solo04*), SW1 (*solo01*), SW2 (*solo12*), and NOAA station 8771341 (Galveston Bay Entrance, North Jetty), during calm conditions (low wind and low waves).

	TT1	TT2	SW1	SW2	NOAA
TT1		1	0.99	0.99	0.97
TT2	2.7		0.98	0.98	0.96
SW1	5.3	4.5		1	0.97
SW2	6.0	4.9	1.2		0.96
NOAA	6.3	5.8	5.7	5.8	

The upper diagonal of the table shows the correlation coefficient (*r*) and the lower diagonal the RMSE in cm.

### Sand temperature and water content

**Fig. 8:**
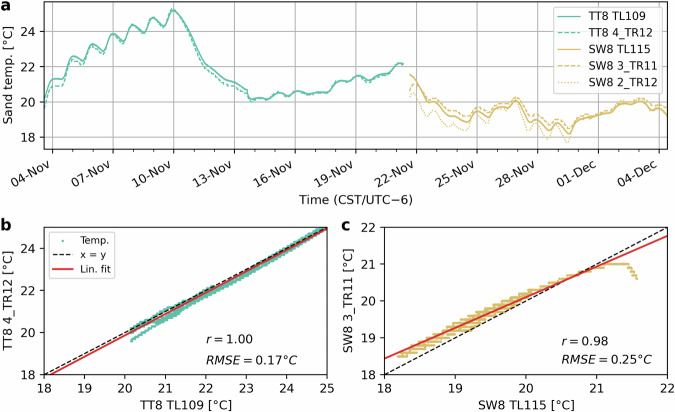
Comparison of the sand temperature readings between the Hobo TLs and the TEROS loggers at the two dune toe stations (TT8 and SW8). (**a**) Time series of sand temperature at TT8 (teal) and SW8 (beige). Panels **b** and **c** show scatter plots of the Hobo TL versus the TEROS temperature data at the two stations, including the RMSE and correlation (*r*).

To assess the reliability of the sand temperature data we compared the readings of the TLs at the dune toe stations (TT8 and SW8) with those of the TEROS loggers at a similar depth. For TT8, this was the TR12 at position 4 (the fourth logger from above), whereas at SW8 the TL was situated roughly in between the TEROS’ at positions 2 and 3. The temperatures agree well with a RMSE of 0.17°*C* at TT8 and 0.25°*C* at SW8 (Fig. 8). The higher RMSE at SW8 makes sense as the TL elevation was in-between two TEROS loggers (we used the TR11 at position 3 to compute the RMSE as it was slightly closer). The errors may be partly caused by the horizontal distance (roughly 1–2 m) between the positions of the loggers. Moreover, at both locations the highest errors occur in the first hours after deploying the TEROS array (the TLs were deployed earlier). The installation of the TEROS array involved digging a deep hole, which stayed open for at least an hour, potentially affecting the sand temperature for several hours after.

We did not have any data to validate the measured water content. The TEROS loggers measure the dielectric permittivity of the soil, which is then converted to the volumetric water content (VWC) through a calibration equation. In the user manual it is claimed that the TEROS 11/12 factory calibration for mineral soils (Eq. (1), where *RAW* is the raw data value) should derive the VWC with an accuracy of approximately 3%^49^. However, for all six loggers, the observed VWC reaches up to 0.6–0.65. Such high values seem unrealistic for sandy beaches, which generally have a porosity in the range of 0.35 to 0.5^61,62^. Unfortunately, we did not calibrate the loggers to site specific soil samples due to time constraints and the mentioned accuracy claim in the user manual. We were therefore unable to correct the observations accordingly. Nonetheless, we make the data accessible, as the relative VWC values and trends thereof may still provide relevant insights into the sand moisture dynamics. Furthermore, the VWC values can be reconverted to the raw dielectric permittivity output using Eq. (1). The raw values can then be converted to VWC again with a different calibration equation. However, the provided VWC should be used with care, taking the above into account.

1 $$VWC=3.879\cdot 1{0}^{-4}\cdot RAW-0.6956$$

### Wave buoys

The National Data Buoy Center (NDBC) operated by NOAA has a buoy (ID *42035*) that records half-hourly offshore wave and wind conditions approximately 37 km east of Galveston^30^. Originally, the plan was to compare the Spotter data to the wave data from NOAA for validation. Unfortunately, buoy *42035* started drifting and stranded on Galveston Island on 25 October 2023, roughly one week before the deployment of the two Spotters. It remained out of service for the entire deployment duration and was only repaired and deployed again in May 2024. This means that there is no verified wave data near the deployed Spotter locations for validation, as the two next closest stations with wave data are NDBC buoy *42019* (≈160 km southwest of Galveston) and NDBC buoy *42091* (≈220 km east of Galveston), both of which are much further offshore than *42035*.

**Fig. 9:**
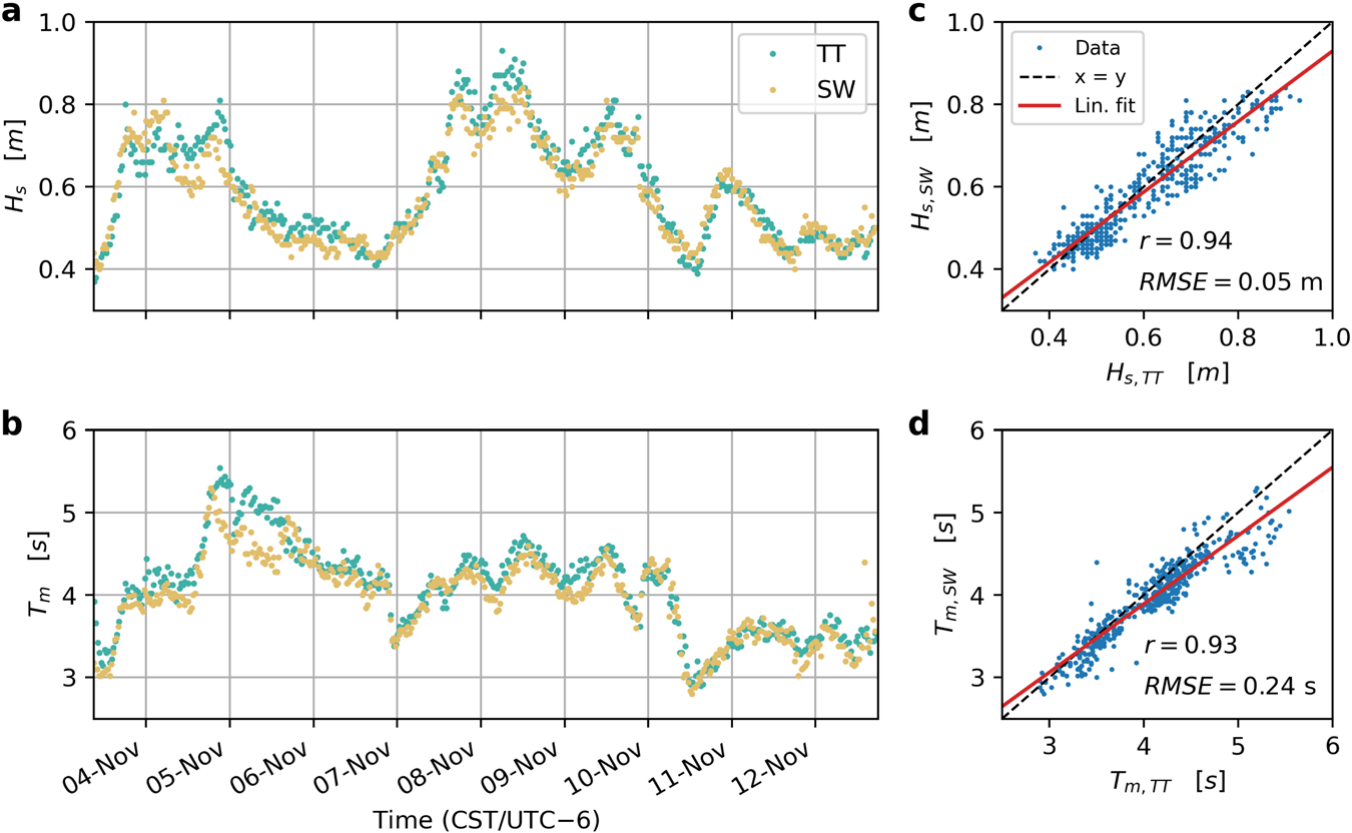
Comparison of the spectral wave data of the two Spotters between 3 and 13 November, showing timeseries of significant wave height (*H*_*s*_, panel **a**) and mean wave period (*T*_*m*_, panel **b**). Panels c and d show scatter plots with correlations and RMSE for *H*_*s*_ (*r* = 0.94, *RMSE* = 0.05 m) and *T*_*m*_ (*r* = 0.93, *RMSE* = 0.24 s) at TT0 and SW0.

Given the simultaneous deployment of the two Spotter buoys in relative proximity to each other (≈13.5 km), we compared the data of the Spotters as a simple validation step. This was only possible for the period between 3 and 12 November, however, before the SW0 Spotter (*spot2*) started drifting. For that validation period, the two Spotters compare well, showing high correlations for significant wave height (*r* = 0.94, *RMSE* = 0.05 m) and mean wave period (*r* = 0.93, *RMSE* = 0.24 s, Fig. 9). Given the lack of further data, we refer to other, more comprehensive validation studies of Spotter wave and wind data^53,63–65^.

### Sediment grain size

**Fig. 10:**
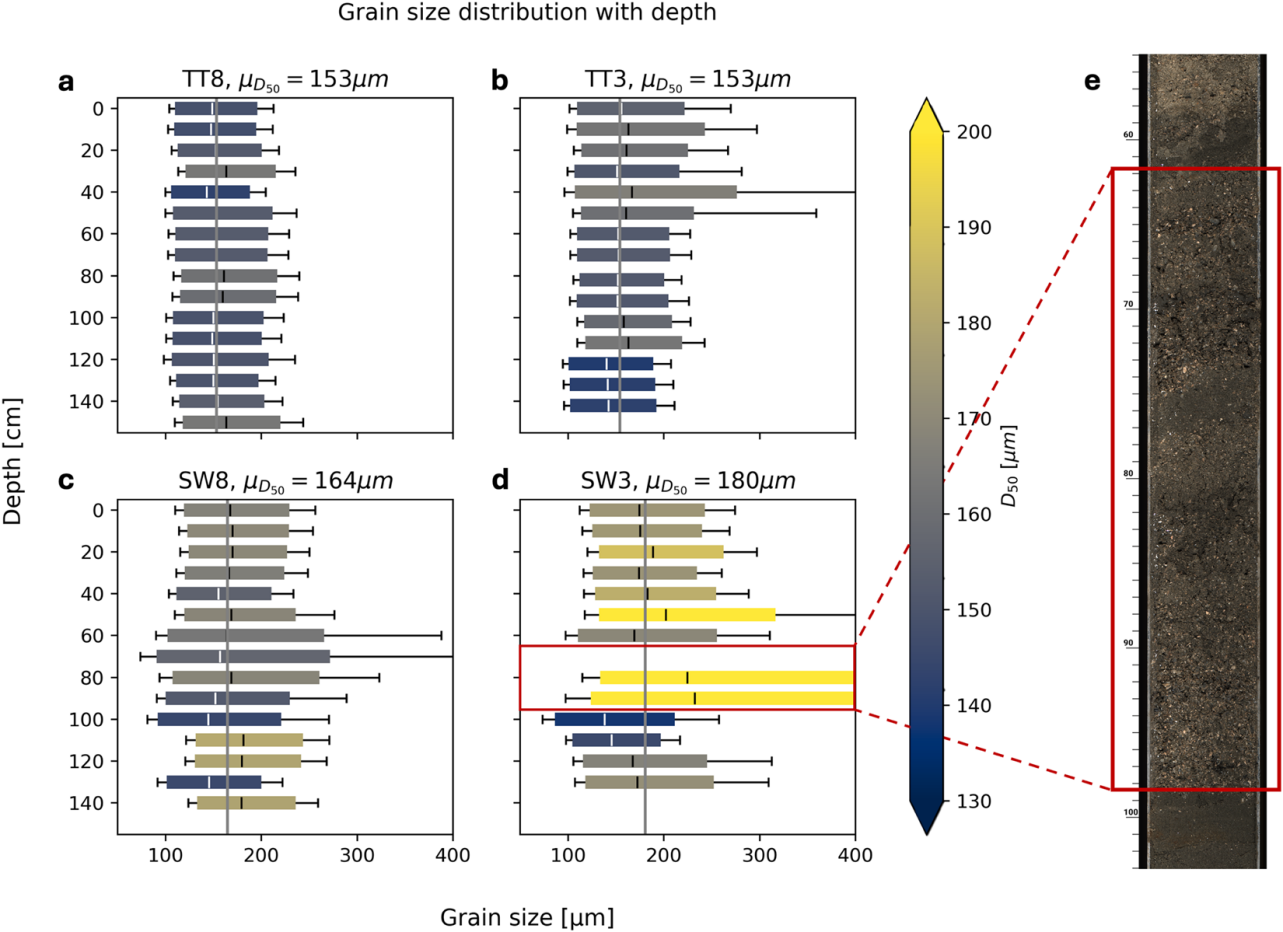
Overview of obtained grainsize distributions for the four cores. The boxplots show the obtained grainsize distributions over the depth of the cores at TT8 (**a**), TT3 (**b**), SW8 (**c**), and SW3 (**d**). The boxplot dimensions show the *D*_50_ (middle line and box color), the range between *D*_20_ and *D*_80_ (extent of the box), and *D*_10_ and *D*_90_ (whiskers). Panel (**e**) shows a snapshot of the linescan from the SW3 core, with the red boxes highlighting the section with predominantly shelly material.

The measured grain sizes at the beach surface showed a mean *D*_50_ of approximately 153 μm at TT and 172 μm at SW (Fig. 10a–d). These results generally lie within the ranges reported in literature^34,35^. The Malvern Mastersizer 2000 is suitable for grain sizes up to 2 mm. While most of the sediment at the two field sites is much finer than that, certain soil layers contained a significant amount of shells/shell fragments larger than 2 mm, especially at SW. These are likely remnants of past nourishments, which were coarser than the native sand and contained shelly material^34^—this also explains the coarser *D*_50_ at SW and most shelly material being found there, as it is much closer to the nourished areas. When preparing the samples for the Malvern we avoided large shell fragments as much as possible, meaning these were not adequately represented in the obtained grain size distributions. However, the core from SW3 contained so much shelly material between 62 and 98 cm from the top (Fig. 10e, marked in red) that we could not prepare completely shell-less samples. Therefore, the derived grain size distribution for these layers may not be well represented by the Malvern results.

## Usage Notes

Usage notes for the specific data files are included in all data folders as *README.txt* files. Most of the data are either stored in NetCDF format (*.nc files) or CSV format (*.csv files). Both file types can be opened and read using most major coding languages. We used Python 3.12 for the processing of all data. If using Python, we recommend *Xarray* for reading NetCDF files and *pandas* for CSV files. The NetCDF files follow CF-1.8 conventions, so when reading them with *Xarray*, the unix time values are automatically converted to datetime objects and missing values (−9999) replaced by *NaN* (*Not a Number*). For any unanswered questions concerning the data or field experiments please contact the authors.

## Data Availability

Python code developed to read out and process the raw data is accessible through the following GitHub repository: https://github.com/jakobchristiaanse/coastal-fieldwork. Additional code can be acquired from the authors upon reasonable request.
